# Insulin-binding protein-5 down-regulates the balance of Th17/Treg

**DOI:** 10.3389/fimmu.2022.1019248

**Published:** 2022-11-01

**Authors:** Mengyuan Zhu, Han Han, Lei Hu, Yu Cao, Zhipeng Fan

**Affiliations:** ^1^Laboratory of Molecular Signaling and Stem Cells Therapy, Beijing Key Laboratory of Tooth Regeneration and Function Reconstruction, Capital Medical University School of Stomatology, Beijing, China; ^2^Department of General Dentistry, Capital Medical University School of Stomatology, Beijing, China; ^3^Research Unit of Tooth Development and Regeneration, Chinese Academy of Medical Sciences, Beijing, China

**Keywords:** inflammation response, inflammatory diseases, insulin-binding protein-5, immune homeostasis, Th17

## Abstract

The inflammatory response plays critical important role in tissue hemostasis. Our previous study showed insulin-binding protein-5 (IGFBP5) could enhance the regeneration of tissue defect under inflammation condition, but the function of IGFBP5 in controlling inflammation and regulating immune responses remains unclear. In present study, we studied the regulatory effect of IGFBP5 on T cell immune response *in vitro*, and the maintenance of Th17/Treg balance *in vivo* by using dextran sulfate sodium salt (DSS)-induced colitis in mice. The results showed that IGFBP5 inhibited the differentiation of CD4^+^ T cells into Th17 subset while promoted its differentiation into Treg subsets. Further results of animal experiments demonstrated that recombinant IGFBP5 reversed the imbalance of Th17/Treg and alleviated the severity of DSS-induced colitis. The percentage of Th17 cells decreased and the percentage of Treg cells increased in the inflamed colon tissue and mesenteric lymph nodes of mice with colitis after IGFBP5 treatment. Besides, pro-inflammatory cytokines such as TNF-α, IL-1β and IFN-γ in serum were suppressed after the treatment of IGFBP5. Moreover, the function of IGFBP5 in regulating Th17/Treg balance could be inhibited by the inhibitors of ERK or JNK pathway. In conclusion, all these data showed that IGFBP5 could regulate Th17/Treg balance *via* ERK or JNK pathways. The findings of our study provide a theoretical basis for the application of IGFBP5 in inflammatory diseases.

## Introduction

The inflammatory response is a defense mechanism that evolved in higher organisms to protect them from infection and injury (1). Its aim is to localize and eliminate the harmful substances and to remove damaged tissue components so that the body can begin to heal (2). Effective inflammatory responses depend on intricate cellular and molecular interactions between the immune system and the tissue. If inflammation is not properly controlled, it can eventually lead to immune-mediated inflammatory diseases, such as periodontitis, rheumatoid arthritis and inflammatory bowel disease (2).

Th17/Treg balance plays a critical role in host defense against extracellular pathogens and resume immune and tissue homeostasis (3, 4). Th17 is important in leading to tissue inflammation and autoimmunity, while Treg prevents autoimmunity and tissue damage resulting from excessive or unnecessary immune activation through their suppressive function (5, 6). The imbalance in Th17/Treg population causes tissue damage and some inflammatory diseases (4) (7). Thus, regulating the balance of Th17/Treg is vital in both the treatment of immune diseases and the repair of damaged tissue.

Recently, immunomodulatory factors such as transforming growth factor β (TGF-β) and prostaglandin E2 (PGE2) have been found to regulate the balance of Th17/Treg (8, 9). Besides, some factors in the process of tissue repair and homeostasis such as BMP, osteopontin and insulin-like growth factor 1 (IGF-1) have also been identified to regulate immune balance, indicating that tissue repair factors which are indirectly related to immunoregulation can also have the potential to regulate immune responses (10–12). The insulin-like growth factor–binding protein (IGFBP) family consists of six structurally similar proteins with high affinity to IGFs and was found to have the ability to enhance tissue regeneration (13, 14). The biological functions of IGFBPs can be categorized as IGFs-dependent and IGFs-independent actions. The IGF-independent actions of IGFBPs include effects on cell adhesion and migration, cell growth and apoptosis (15, 16). IGFBP5 is one of the six IGFBPs, which were characterized by binding to IGF-I and has been shown to have IGF-independent activities (13, 17, 18). Our previous study found that IGFBP5 had anti-inflammation potentials of MSCs and promoted periodontal tissue regeneration in minipig (19, 20). Several studies have shown that IGFBP5 have direct influence on inflammation mediated by some immunocytes, like mononuclear cells or CD4^+^ T cells (21) ^(^ 22^),^, the detailed function of IGFBP5 in controlling inflammation remains unclear.

In this study, the functions of IGFBP5 on T cell subsets were evaluated *in vitro*, and the effect of IGFBP5 on immune Th17/Treg homeostasis in a DSS-induced colitis model of mice was assessed *in vivo*. The results demonstrated that IGFBP5 had an anti-inflammatory effect *via* maintaining immune homeostasis and could efficiently attenuated the severity of DSS-induced colitis, providing an immunological support for the control of inflammation and the treatment of other immune-mediated inflammatory diseases.

## Materials and methods

### Animals and ethics

The study was approved by the Animal Care and Use Committee at the Beijing Stomatological Hospital, Capital Medical University (Ethics Review No. KQYY-202008-004). We use the animals in accordance with the guidelines of the Experimental Animal Management Ordinance. Six-week-old male C57BL/6 mice were bought from Beijing Vital River Laboratory Animal Technology Co., Ltd.

### Induction of UC model and treatment

To explore the role of IGFBP5 in the occurrence and progression of experimental UC in mice, the animals were randomly divided into three groups (5 mice in each group): control group, dextran sulfate sodium (DSS, MP Biomedicals, Santa Ana, CA, USA)-induced experimental UC group (DSS+PBS group) and recombinant mouse IGFBP5 protein (578-B5-025, R&D system, USA) treatment group (DSS+IGFBP5 group). 7 days of 3% (w/v) DSS solution was used to induce UC model, and IGFBP5 (0.5μg per mouse at a time) was intraperitoneal injected on day 3 and day 5 to assess the therapeutic potential of IGFBP5.

### Disease activity index scoring

The mice in each group were weighed and recorded at a fixed time every day from the beginning to the end of the experiment. The shape of stool and the degree of hematochezia of each mouse were observed and recorded, and the DAI score of each mouse was obtained according to the DAI standard (1). Weight loss scoring criteria: score 0, no weight loss; score 1, 1-5% weight loss; score 2, 5-10% weight loss; score 3, 10-15% weight loss; score 4, more than 15% weight loss. (2) Scoring criteria of stool viscosity: score 0, normal; score 1, stool forming slightly soft; score 2, loose stool; score 3, diarrhea; score 4, dysentery-like stool. (3) Scoring criteria of fecal occult blood: score 0, fecal occult blood negative; score 1, fecal occult blood positive; score 2, visible blood in the stool; score 3, gross bloody stool; score 4, rectal bleeding. DAI scoring: the average of the sum of the above three scores.

### Total stool DNA extraction and 16S rDNA gene high-throughput sequencing

Database sequencing: After the total DNA of fresh feces was extracted, the specific primers was synthesized according to the full-length sequence of primers. PCR amplification, product purification, quantification, homogenization and sequencing database (SMRT Bell) formation were carried out. The database was first inspected, and the qualified database was sequenced by PacBio Sequel.

The 16S rDNA sequencing data were analyzed on the BMKCloud platform (https://international.biocloud.net). The third-generation microbial diversity is based on the PacBio sequencing platform, and the marker genes are sequenced by single-molecule real-time (SMRT Cell). Then the circular consensus sequencing (CCS) was filtered, clustered or denoised, and the species annotation and abundance analysis were carried out to identify the species composition of the samples. Further Alpha Diversity was assessed according to community diversity and richness based on the operational taxonomic units (OUT) number with the ACE, Chao, Shannon and Simpson index. Student’s t-test was utilized to discover the differences among the groups. Beta diversity was determined by principal component analysis (PCA) based on the distance matrix.

### Histological examination and histological damage index

The colon tissues were collected, fixed with 4% paraformaldehyde, dehydrated, and embedded into paraffin. Then, the tissues were sliced into 5μm and stained with hematoxylin and eosin (H&E) to assess the inflammation of colon. Images were captured under microscope. HAI score was used to evaluate the degree of colon injury and inflammatory infiltration. According to Rachmilewitz et al., colon pathology was graded by four parameters, including neutrophil margination and tissue infiltration, hemorrhagic congestion and edema of the mucosa, the goblet cell depletion, and the crypt loss. Based on the degree of pathological lesion of colons, each parameter was divided into 0-4 grades, and those with no change of colon were rated as level 0 and scored 0. Those with minor changes were grade 1 and scored 1 point; those with slight changes were grade 2 and scored 2 points; those with moderate changes were grade 3 and scored 3 points; those with severe changes were grade 4 and scored 4 points. A minimum score of 0 indicates no inflammatory damage, while a higher score indicates more inflammatory damage to colons.

### Cell culture and flow cytometry

Mesenteric lymph nodes were taken from each group of mice and incubated in buffer solution (PBS containing 0.5% BSA). The lymph nodes were placed on a 70μm filter, washed several times with buffer solution, centrifuged at 1500rpm for 6 minutes. The supernatant was discarded, and the total cells of lymph nodes were suspended in complete RPMI 1640 medium containing 10% fetal bovine serum (FBS), 1% penicillin and streptomycin (Gibco), 10mM HEPES (Gibco), 2mM L-Glutamine (Gibco), 50nM 2-Mercaptoethanol (Gibco), 1mM sodium pyruvate (Sigma-Aldrich), and 100μM MEM non-essential amino acids (Sigma-Aldrich) at 37°C with 5% CO_2_. For *in vitro* experiments, cells were coated with PBS containing anti-CD3ϵ (1μg/mL, 100302, Biolegend, USA) overnight at 4°C one day before cell isolation. 3×10^6^ naive CD4^+^ T cells were isolated from a single-cell suspension of 6×10^7^ lymphocytes from mesenteric lymph nodes of six mice by Naive CD4^+^ T Cell Isolation Kit (130-104-453, Miltenyi Biotec, Germany), LS columns (130-042-401, Miltenyi Biotec, Germany) and a MidiMACS™ separator. Before adding T cell suspension, discard PBS and add anti-CD28 (102102, Biolegend, USA) with a final concentration of 2μg/mL into the medium. PD98059 (20μM, #9900S, Cell Signaling Technology, USA) and SP600125 (20μM, HY-12041, MCE, USA) were added 1h before the treatment with IGFBP5 to inhibit the activation of ERK and JNK pathways. Cells were treated with recombinant IGFBP5 protein for 3 days. For *in vivo* studies, mice were anaesthetized and sacrficed on the 7^th^ day, and total cells of mesenteric lymph nodes were collected for further fluorescence staining.

After excluding cell debris and dead cells, the remaining cells were fixed and fluorescently stained with FITC anti-mouse CD4 antibody (100406, Biolegend, USA) and APC anti-mouse CD25 antibody (101910, Biolegend, USA) for Tregs, anti-CD4 antibody and PE anti-mouse IL-17A antibody (506904, Biolegend, USA) for Th17, anti-CD4 antibody and APC anti-mouse IFN-γ antibody (505810, Biolegend, USA) for Th1, and anti-CD4 antibody and PE anti-mouse IL-4 antibody (504104, Biolegend, USA) for Th2, and analyzed by flow cytometry.

### Immunohistochemistry analysis

Colon tissues were collected and fixed with 4% paraformaldehyde, dehydrated and embedded into paraffin. Antigens were retrieved by heating at 95° C in 0.1M citrate buffer for 15 minutes, and then incubated in endogenous peroxidase enzymes blocking buffer for 10 minutes at room temperature. Tissue sections were blocked with PBS containing 5% BSA and 1% Triton X-100 (T8200, Solarbio Life Science, China) for 30 minutes at room temperature, and stained with IGFBP5 (1:200, 55205-1-AP, proteintech, China), FOXP3 (1:400, #12653S, Cell Signaling Technology, USA) and RORγt (1:500, bs-23110R, Bioss, China) primary antibodies at 4°C overnight. Next, tissues were incubated in goat anti-rabbit/mouse IgG and streptavidin-HRP separately for 10 minutes. Finally, staining was carried out using DAB solution (CW2069S, Cwbio, China). All images were taken under the same exposure and intensity settings.

### Enzyme linked immunosorbent assay

For the ELISA of cytokines, the serum samples of mice were collected, clot at room temperature for 30 minutes, and centrifuged at 3000 rpm for 10 minutes at 4°C. Then, the samples were transferred into 96-well ELISA plates. The concentrations of TNF-α, IL-1β and IFN-γ were assessed by mouse TNF-α precoated ELISA kit (Cat#: 1217202, Dakewei Bio-engineering, China), mouse IL-1β precoated ELISA kit (Cat#: 1210122, Dakewei Bio-engineering, China), and mouse IFN-γ precoated ELISA kit (Cat#: 1210002, Dakewei Bio-engineering, China) according to the manufacturer’s instruction.

### Real‐time reverse transcriptase‐polymerase chain reaction

Total RNA of normal and colitis tissue *in vivo* and CD4^+^ T cells *in vitro* were isolated by Trizol reagent (Invitrogen). 2μg of total RNA was reverse-transcribed into cDNA with oligo (dT) or random primers, according to the manufacturer’s instruction (Invitrogen). Real-time RT-PCR procedures were performed with QuantiTect SYBR Green PCR kit (Qiagen, Hilden, Germany). The iCycleriQ Multicolor Real‐time PCR Detection System was employed for real‐time RT‐PCR. The primers used in the experiment were listed in **Table 1**. Relative quantification of gene expression was performed by using the 2^-ΔΔCt^ method in which the expression of each target gene was normalized to the housekeeping gene glyceraldehyde3-phosphate dehydrogenase.

**Table 1:** Primers used in the real-time PCR assays.

Genes	Primer	Primer Sequences (5’→3’)
*Gapdh*	Forward primer	AGGTCGGTGTGAACGGATTTG
	Reverse primer	TGTAGACCATGTAGTTGAGGTCA
*Igfbp5*	Forward primer	CCCTGCGACGAGAAAGCTC
	Reverse primer	GCTCTTTTCGTTGAGGCAAACC
*RORγt*	Forward primer	CTCTTTTCACGGGAGGAGGT
	Reverse primer	CTCCATGAAGCCTGAAAGCC
*Foxp3*	Forward primer	ACCATTGGTTTACTCGCATGT
	Reverse primer	TCCACTCGCACAAAGCACTT
*T-bet*	Forward primer	CTCAGGACTAGGCGAAGGAG
	Reverse primer	ACTGGCCTTCGGTTTCCTTA
*Gata3*	Forward primer	CCCCATTACCACCTATCCGC
	Reverse primer	CCTCGACTTACATCCGAACCC
*Ki-67*	Forward primer	GCTCTCTTTAACTCAGCGCC
	Reverse primer	TCTCAGGCTTGCTGAAGGAA
*CD25*	Forward primer	CAAGAACGGCACCATCCTAAA
	Reverse primer	TCCTAAGCAACGCATATAGACCA
*CD44*	Forward primer	CATCCCAACGCTATCTGTGC
	Reverse primer	ATAGTGGGAGGTGTTGGACG
*CD69*	Forward primer	AAGCGATATTCTGGTGAACTGG
	Reverse primer	ATTTGCCCATTTCCATGTCTGA
*IL-2*	Forward primer	AGCAGCTGTTGATGGACCTA
	Reverse primer	CTGGGGAGTTTCAGGTTCCT

### Statistical analysis

All data were shown as mean ± standard error of the mean (SEM). Statistical calculations were analyzed by GraphPad Prism version 7.0 software and statistical significance was determined by Student’s t-test or one-way ANOVA analysis, and *p*<0.05 was considered statistically significant.

## Results

### IGFBP5 expression reduced in mice with DSS-induced colitis

To explore the changes of IGFBP5 in mice with DSS-induced colitis, IHC and RT-qPCR were performed, respectively. As can be seen in **Figure S1A**, IGFBP5 mainly expressed inside of the intestinal epithelium in normal colon tissue, which reduced in mice with DSS-induced colitis at the site of inflammation. Moreover, the results of RT-qPCR showed that the expression of *Igfbp5* decreased in mice with DSS-induced colitis (**Figure S1B**). These results revealed that IGFBP5 might be important in the pathogenesis of colitis.

### IGFBP5 increased the percentage of CD4^+^ CD25^+^ Treg and decreased the percentage of CD4^+^ IL-17A^+^ Th17 *in vitro*

**Figure 1:**
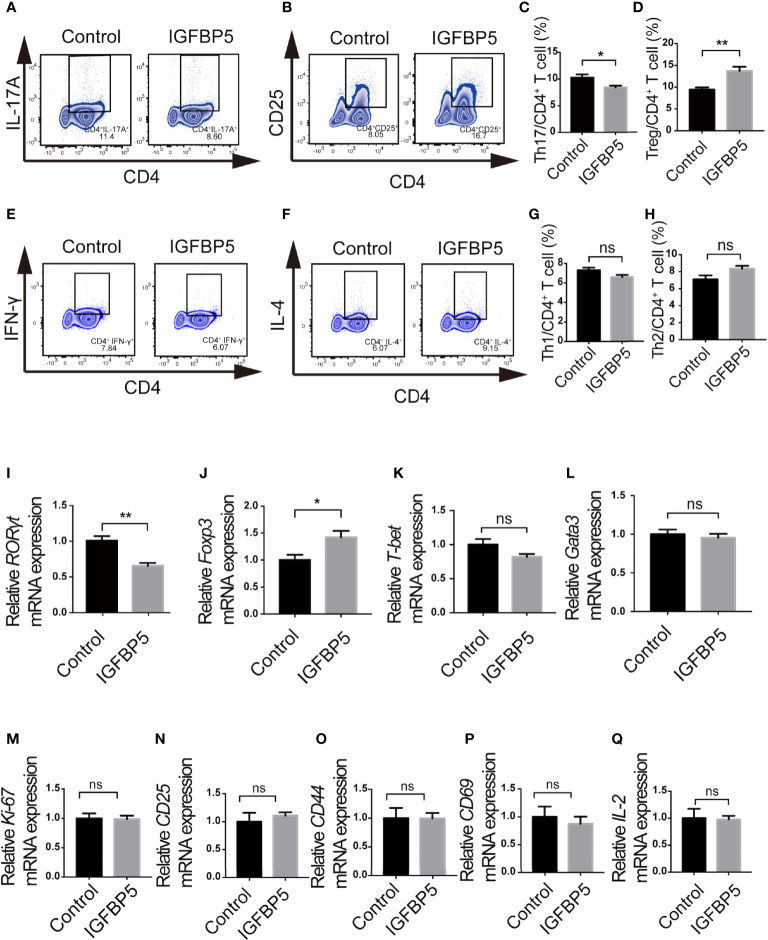
IGFBP5 increased the percentage of Treg cells and decreased the percentage of Th17 cells. Flow cytometry and RT-qPCR were used to detect the effect of IGFBP5 on T cell subsets *in vitro*. **(A, C)** Flow cytometry showed that the percentage of CD4^+^ IL-17A^+^ Th17 cells in the CD3/CD28 activated T cells decreased after the treatment of recombinant IGFBP5. **(B, D)** Flow cytometry showed that the percentage of CD4^+^ CD25^+^ Treg cells in the CD3/CD28 activated T cells increased after IGFBP5 treatment. **(E, G)** Flow cytometry showed that the percentage of CD4^+^ IFN-γ^+^ Th1 cells in the CD3/CD28 activated T cells decreased slightly after the treatment of recombinant IGFBP5. **(F, H)** Flow cytometry showed that the percentage of CD4^+^ IL-4^+^ Th2 cells in the CD3/CD28 activated T cells increased slightly after IGFBP5 treatment. **(I, J)** RT-qPCR showed that the expression of *RORγt* was significantly reduced and the expression of *Foxp3* was markedly increased in IGFBP5 group. **(K, L)** RT-qPCR showed that the expression of *T-bet* was slightly reduced and there was little effect on the expression of *Gata3* after the treatment of IGFBP5. **(M-Q)** The relative mRNA levels of *Ki-67* and activation-related *CD25*, *CD44*, *CD69* and *IL-2*. Student’s t-test was used to determine statistical significance. The data are presented as the mean ± SEM (n=3). The experiment was repeated three times independently. *p < 0.05, **p < 0.01; ns, not significant.

We designed an *in vitro* experiment to detect the effect of recombinant IGFBP5 on T cell differentiation in the adaptive immune response. T cells were stained with fluorochrome-conjugated antibodies against CD4, CD25, IFN-γ, IL-4, and IL-17A, and the changes in T cell subsets levels were examined by flow cytometry. The results of flow cytometry showed that the percentage of CD4^+^ CD25^+^ Treg cell subset in the CD3/CD28 activated T cells treated with recombinant IGFBP5 significantly increased (**Figures 1B, D**), while the percentage of CD4^+^ IL-17A^+^ Th17 cell subset decreased significantly compared with the control group (**Figures 1A, C**). In addition, the percentage of CD4^+^ IFN-γ^+^ cells were slightly decreased and the percentage of CD4^+^ IL-4^+^ Th2 cell subset was slightly increased in IGFBP5-treated group, compared with those in the control group (*p*>0.05, **Figures 1E-H**). These results demonstrated that recombinant IGFBP5 specifically increases the percentage of Treg cell subsets and decreases the percentage of Th17 subsets, but not other immune cell populations, which may affect immune-inflammatory responses *in vivo*.

Furthermore, RT-qPCR was used to assess the level of *T-bet*, *Gata3*, *RORγt* and *Foxp3*, which are key transcription factors of Th1, Th2, Th17 and Treg subsets, respectively, to determine whether recombinant IGFBP5 can affect T cell subsets. The results of relative mRNA expression showed that after the treatment of recombinant IGFBP5 on CD3/CD28 activated T cells isolated from the lymph nodes of mice, the expression of *RORγt* was significantly down-regulated (**Figure 1I**) and the expression of *Foxp3* was significantly up-regulated (**Figure 1J**) compared to the control group. Besides, both the expression of *T-bet* and *Gata3* was slightly down-regulated (*p*>0.05, **Figures 1K, L**). **Figures 1M–Q** showed that the proliferation (Ki-67) ability or activation capacity (CD25, CD44, CD69 and IL-2) of CD4^+^ T cells were not significantly affected by IGFBP5 (p>0.05).

### IGFBP5 alleviated dextran sulfate sodium-induced colitis

**Figure 2:**
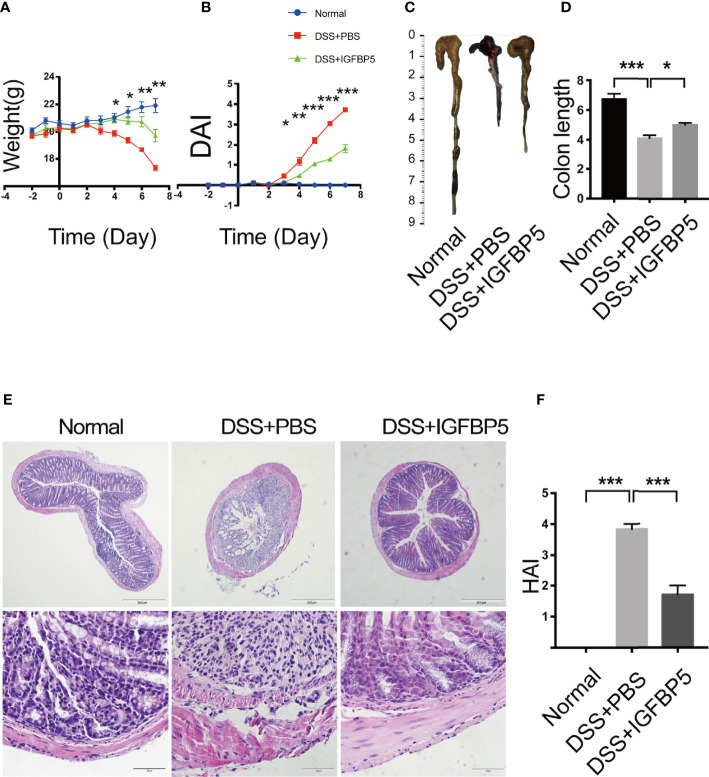
IGFBP5 ameliorated DSS-induced colitis in mice. **(A)** Daily body weight during the experiment. **(B)** Disease activity index (DAI) every day during the experiment. **(C)** Representative photo of the colon in each group and **(D)** Quantitative analysis of colon length in each group (n=5). **(E)** Hematoxylin and eosin (H&E) staining of colon sections were examined after the mice were sacrificed (4× and 20×). **(F)** Histological damage index (HAI) was determined. One-way ANOVA was used to determine statistical significance. The data are presented as the mean ± SEM (n=5). The experiment was repeated three times independently. *p < 0.05, **p < 0.01, ***p < 0.001.

3% DSS was given to the mice as drinking water for 7 days to induce UC. On the 3^rd^ and 5^th^ day of the experiment, recombinant IGFBP5 was intraperitoneal injected twice. To explore the effect of IGFBP5 on the development of colitis, we evaluated the clinical scores according to weight loss, shape of stool and the degree of hematochezia. Since colon in mice with colitis would be significantly shortened, we also recorded the colon length. As can be seen in **Figure 2A**, the weight of mice in DSS+PBS group reduced gradually as time went by. When treated with IGFBP5, weight loss in mice was alleviated. The difference between DSS+PBS group and DSS+IGFBP5 group was statistically significant since day 4. Besides, mice with DSS-induced colitis would have loose and bloody stool. IGFBP5 treatment significantly relieved the symptoms of colitis. The DAI scoring of mice treated with IGFBP5 was lower than that of mice treated with PBS (**Figure 2B**). After the mice were sacrificed on the 7^th^ day, we photographed and measured the length of colon of each mouse. The results indicated that the colon length of mice in DSS+IGFBP5 group was longer than that of mice in the other groups (**Figure 2C**). The results of quantitative analysis are shown in **Figure 2D**.

The results of histological examination appeared that the colon tissue of the mice in control group was intact, and the glands and epithelial cells were neatly arranged, while the colon tissue of the mice in DSS+PBS group was incomplete, with large areas of ulcers and the continuity of mucosa was destroyed. The glands were extremely irregular, goblet cells decreased, and abundant inflammatory cells infiltrated into the lamina propria (**Figure 2E**). Compared with DSS+PBS group, the destruction of colonic mucosa in mice treated with recombinant IGFBP5 was alleviated, and damaged mucosa coexisted with intact mucosa, with decreased infiltration of inflammatory cells.

The histological damage index (HAI) of mice in each group was shown in **Figure 2F**. The HAI of mice in DSS+PBS group was higher than that of control group, and it decreased after the treatment of recombinant IGFBP5. Taken together, intraperitoneal injection of recombinant IGFBP5 could alleviate colon damage of mice with UC induced by DSS.

### IGFBP5 maintained the balance of Th17/Treg cells in the mesenteric lymph nodes

**Figure 3:**
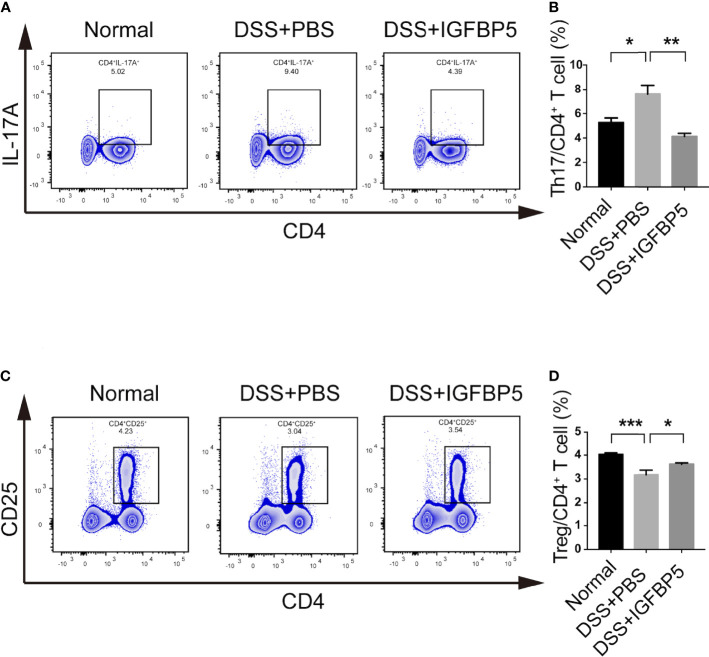
IGFBP5 maintained the balance of Th17/Treg cells in the mesenteric lymph nodes of mice. Cells were isolated from the mesenteric lymph nodes of mice with DSS-induced colitis and stained with fluorochrome-conjugated antibodies against CD4, CD25 and IL-17A. **(A)** The number of Th17 were expressed as percentages of the CD4^+^ IL-17A^+^ cell population. **(B)** Statistical analysis of Th17. **(C)** The number of Tregs were expressed as percentages of the CD4^+^ CD25^+^ cell population. **(D)** Statistical analysis of Tregs. The experiment was repeated three times independently. One-way ANOVA was used to determine statistical significance. The data are presented as the mean ± SEM (n=5). *p < 0.05, **p < 0.01, ***p < 0.001.

After extracting the mesenteric lymph node tissue of mice in each group, we detected the cells by flow cytometry. Treg cells are one of the important subsets of CD4^+^ lymphocytes, which are linked to the regulation of immune homeostasis and tolerance. Additionally, the balance of Th17/Treg is significant for both the development and recovery of UC. To explore whether IGFBP5 treatment affects the balance of Th17/Treg cells, we detected the percentage of Th17 and Treg cells in mesenteric lymph nodes. The results revealed that the percentage of Th17 (CD4^+^ IL-17A^+^) cells in the mesenteric lymph nodes of mice in DSS+PBS group was higher than that in normal group, and higher compared with that in DSS+IGFBP5 group (**Figures 3A, B**). Besides, the percentage of CD4^+^ CD25^+^ Treg cells in the mesenteric lymph nodes of mice with DSS-induced colitis was lower than that in normal group, while the percentage of CD4^+^ CD25^+^ Treg cells increased by recombinant IGFBP5 treatment (**Figures 3C, D**). To sum up, recombinant IGFBP5 treatment could maintain the balance of Th17/Treg cells in mesenteric lymph nodes of mice with DSS-induced colitis.

### IGFBP5 maintained the balance of mucosal Th17/Treg in the inflamed colon

**Figure 4:**
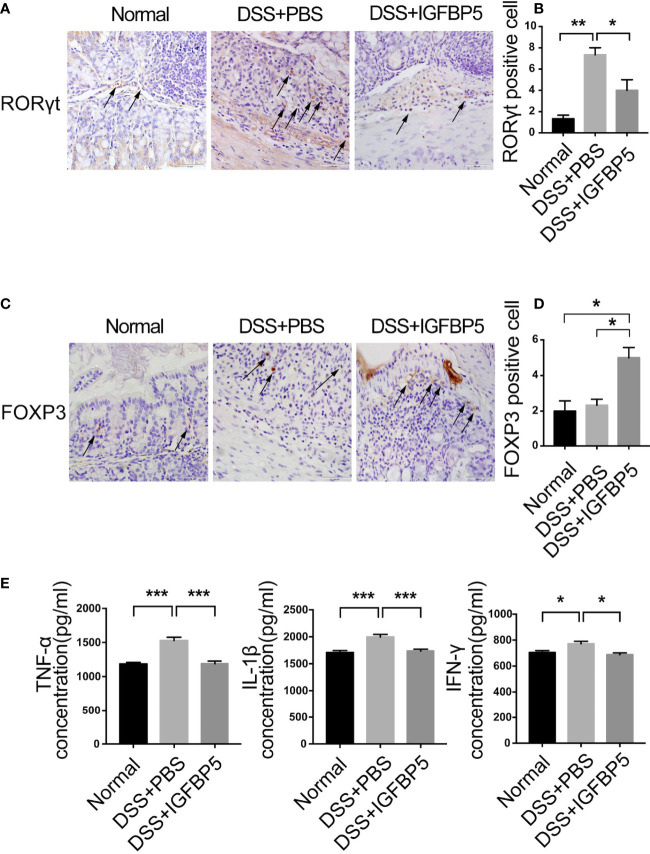
IGFBP5 maintained the balance of mucosal Th17/Treg in the inflamed colon. The expression of **(A)** RORγt and **(C)** FOXP3 in colon tissue were detected by immunohistochemistry (4× and 20×), and the quantitative analysis of RORγt and FOXP3 positive cells were shown respectively, in **(B)** and **(D)**. **(E)** The concentrations of TNF-α、; IL-β and IFN-γ in the serum of mice in each group were determined by ELISA (n=5). The experiment was repeated three times independently. One-way ANOVA was used to determine statistical significance. The data are presented as the mean ± SEM. *p < 0.05, **p < 0.01, ***p < 0.001.

Immunohistochemical staining and quantitative measurements results revealed a weak expression of RORγt in the normal group and DSS+IGFBP5 group (**Figures 4A, B**). However, the DSS+PBS group had a strong expression of RORγt in lamina propria. In contrast, there was a strong expression of FOXP3 in the colon tissue of DSS+IGFBP5 group than in the control group and DSS+PBS group (**Figures 4C, D**). Thus, it is indicated that the number of Th17 cells increased in the colon of UC mice, and recombinant IGFBP5 treatment reduced the infiltration of Th17 cells while increased the protective Treg cells.

In UC, the concentrations of pro-inflammatory cytokines, TNF-α, IL-1β and IFN-γ in serum increased predictably. To investigate whether IGFBP5 can regulate TNF-α, IL-1β and IFN-γ production in DSS-induced colitis in mice, ELISA was conducted to explore the concentrations of these cytokines. As shown in **Figure 4E**, in DSS+PBS group, TNF-α, IL-1β and IFN-γ appeared higher levels of expression than the control group. After recombinant IGFBP5 treatment, the levels of pro-inflammatory cytokines decreased compared with DSS+PBS group.

### IGFBP5 regulated the balance of Th17/Treg partly through ERK and JNK pathways

**Figure 5:**
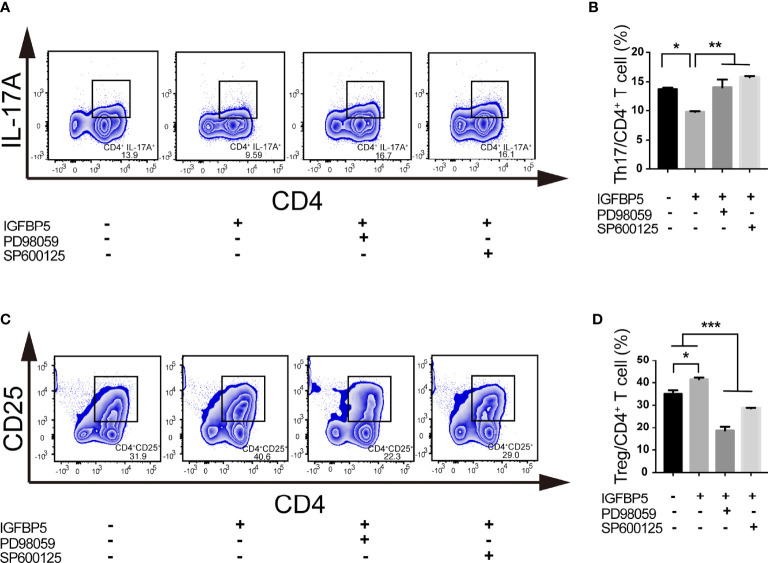
IGFBP5 regulated the balance of Th17/Treg partly through ERK and JNK pathways. Flow cytometry was used to investigate the key mechanisms of IGFBP5-mediated Th17/Treg ratio change under the pretreatment of ERK and JNK pathway inhibitors, PD98059 and SP600125, before IGFBP5 treatment. **(A, B)** Flow cytometry showed that the percentage of CD4^+^ IL-17A^+^ Th17 cells in the CD3/CD28 activated T cells increased with the presence of PD98059 and SP600125. **(C, D)** Flow cytometry showed that the percentage of CD4^+^ CD25^+^ Treg cells in the CD3/CD28 activated T cells decreased with the presence of PD98059 and SP600125. The experiment was repeated three times independently. One-way ANOVA was used to determine statistical significance. The data are presented as the mean ± SEM (n=3). *p < 0.05, **p < 0.01, ***p < 0.001.

To investigate the key mechanisms of IGFBP5-mediated Th17/Treg ratio change, we detected the change of the percentage of Th17 and Treg cells under the pretreatment of PD98059 and SP600125, the inhibitors of ERK and JNK pathway, before IGFBP5 treatment. The results of flow cytometry showed that after the treatment of PD98059 and SP600125, the differentiation of Th17 cells was promoted and the differentiation of Treg cells was suppressed (**Figures 5A-D**). These results indicated that IGFBP5 had the function of regulating Th17/Treg balance *via* ERK and JNK pathways.

### IGFBP5 regulated gut microbiota in DSS-induced colitis

**Figure 6:**
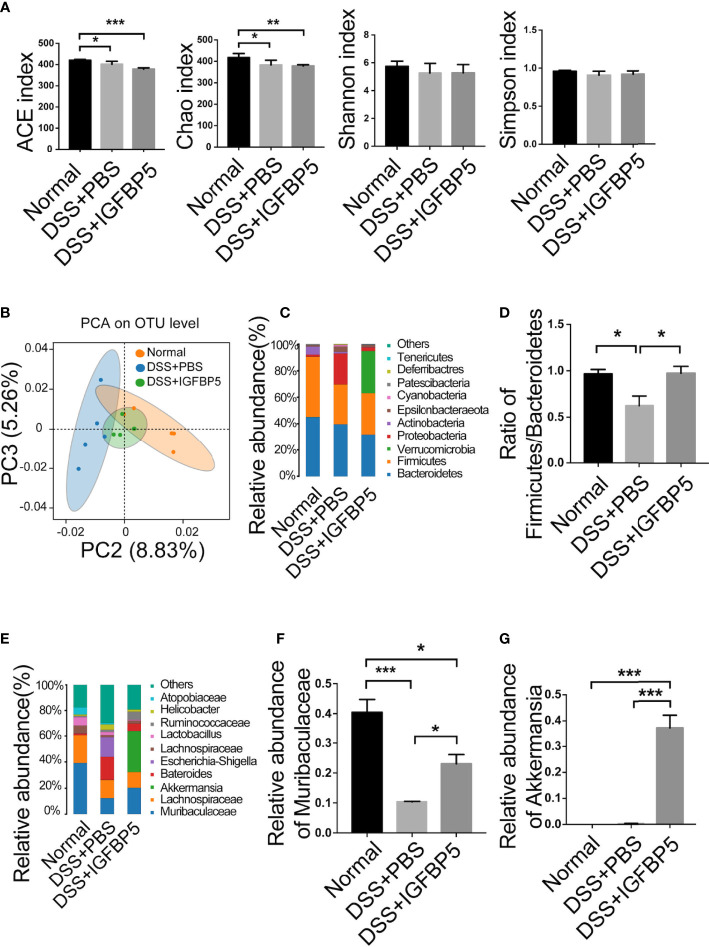
IGFBP5 regulated gut microbiota in DSS-induced colitis. Fresh stools were collected when the mice were sacrificed on Day 7, and DNA was extracted for 16S rDNA sequencing. The alpha diversity index of the gut microbiota was shown in **(A)** ACE, Chao, Shannon and Simpson index. **(B)** PCA of the intestinal microbiota at the OTU level in mice. PC2 and PC3 exhibited 8.83% and 5.26% of the variation, respectively. **(C)** Relative abundance of bacteria at phylum levels (>1%) in Normal, DSS+PBS and DSS+IGFBP5 group. **(D)** The Firmicutes/Bacteroidetes ratio at the phylum level. **(E)** Relative abundance of bacteria at genus levels (>1%) in Normal, DSS+PBS and DSS+IGFBP5 group. **(F)** Relative abundance of Muribaculaceae at genus levels in each group. **(G)** Relative abundance of Akkermansia at genus levels in each group. One-way ANOVA was used to determine statistical significance. The data are presented as the mean ± SEM (n=5) for each treatment. *p < 0.05, **p < 0.01, ***p < 0.001.

To study whether IGFBP5 can inhibit inflammation by regulating the intestinal flora, 16S rRNA high-throughput sequencing was used to analyze fecal samples. As can be seen in **Figure 6A**, the ACE and Chao index in the DSS+PBS group decreased. Compared with DSS+PBS group, the influence of ACE and Chao index in DSS+IGFBP5 group was not significant, which indicated that alpha diversity in DSS+IGFBP5 group was lower than that in the normal group, and there was no significant difference between DSS+IGFBP5 group and DSS+PBS group (*p*>0.05). Shannon and Simpson index were also used to assess microbial diversity of samples. The higher the values of Shannon index and Simpson index, the higher the species diversity of the samples. As shown in **Figure 6A**, Shannon and Simpson index in DSS+PBS group and DSS+IGFBP5 group were slightly lower than those in normal group (*p*>0.05), indicating that DSS stimulation to some extent decreased the diversity of intestinal flora of mice. At the OUT level, principal component analysis (PCA) revealed the overall structure of the intestinal microbiota in the different groups. The normal group, DSS+PBS group and DSS+IGFBP5 group all showed some degree of separation (**Figure 6B**), identifying the variation upon IGFBP5 treatment.

In addition, as two dominant bacteria of intestinal flora at phylum level, the imbalance of the Firmicutes/Bacteroidetes ratio is related to many diseases, and one of the characteristics of intestinal flora in patients with inflammatory bowel disease (IBD) is the decrease of this ratio. The Firmicutes/Bacteroidetes ratio was lower in DSS+PBS group than in normal group, while in DSS+IGFBP5 group, it remained almost the same as the normal group (**Figures 6C, 6D**), indicating that IGFBP5 treatment can restore the balance of some intestinal flora. At genus level, the predominant bacteria in normal group were Muribaculaceae. It is shown that the relative abundance of Muribaculaceae in DSS+PBS group and DSS+IGFBP5 group was lower than that of normal group, among which the lowest was that in DSS+PBS group, which had a significant difference between DSS+IGFBP5 group (**Figures 6E, F**). In DSS+IGFBP5 group, Akkermansia became the dominant bacteria, which was significantly higher than the other two groups (**Figures 6E, G**). The increase of the number of Akkermansia contributes to alleviating the symptoms of inflammatory bowel disease.

## Discussion

Studies have shown that the IGFBPs family has a direct effect on inflammation and immunity (13, 22). Our previous studies found that IGFBP5 could facilitate osteogenic and odontogenic differentiation of MSCs (14). However, the regulatory role of IGFBP5 on local inflammation remains unclear. T cells, especially T helper 17 cells (Th17 cells), which are distinct from the classical Th1 and Th2 cell subsets, play critical roles in the pathogenesis of immune-mediated inflammatory diseases (23, 24) (25),. Interleukin−17 (IL−17) is a pro-inflammatory cytokine that contributes to the pathogenesis of several inflammatory diseases (26). A major source of IL−17 is Th17 cells. IL−17 and Th17 cells are considered to be potential targets for the treatment of several inflammatory and autoimmune diseases (5). DSS-induced ulcerative colitis (UC), as an immune-mediated inflammatory disease mainly dependent on Th17 cells, is a typical disease model to study immune homeostasis (27). The processes of IL-17 in UC have some associations with Periodontitis (28). A study reported a significantly higher prevalence of periodontitis in patients with UC compared to non-UC individuals (29). Results of clinical trials suggest that IL−17 inhibition could be beneficial for the treatment of chronic inflammatory diseases, including periodontitis (30, 31). Currently, the treatment of periodontitis cannot achieve perfect inflammation control and periodontal tissue regeneration (32) (33),. Interestingly, we discover the expression difference of IGFBP5 between normal and colitis tissue using the typical inflammatory immune model-DSS induced experimental UC model in mice. To further enhance the feasibility of IGFBP5 in the control of inflammation and in the treatment of systemic or local immune diseases, especially mediated by IL-17. Then, we explored the regulatory impact and the possible mechanisms of IGFBP5 on host immune homeostasis, providing a theoretical basis for the application of IGFBP5 in the treatment of inflammatory diseases.

Our *in vitro* experiments proved that IGFBP5 could affect the differentiation ability of CD4^+^ T cells under inflammatory stimulation, but not the proliferation or activation capacity (see **Figure 1**). Specifically, IGFBP5 could down-regulate the proportion of Th17 subset and up-regulate the proportion of Treg cells in CD4^+^ T cells stimulated by anti-CD3 and anti-CD28. This indicated IGFBP5 regulate the balance of CD4^+^ T cells into Th17/Treg differentiation. Our previous studies showed IGFBP5 activated multiple signaling pathways to achieve MSCs therapy. For example, IGFBP5 enhances osteogenic differentiation potential of PDLSCs and dentinogenesis potential of dental pulp stem cells (DPSCs) through the JNK and MEK/Erk signaling pathways (14, 20). Recombinant human IGFBP5 protein increased the expression of phosphorylated c-Jun N-terminal kinase (p-JNK), phosphorylated mitogen-activated protein kinase 1 and 2 (p-MEK1/2) as well as phosphorylated extracellular regulated protein kinases (p-Erk1/2) in PDLSCs and DPSCs (14, 20). Evidence have showed these pathways could play roles in regulating the expression of RORγt and Foxp3. The differentiation ability and functions of Th17 cells in allergic rhinitis are inhibited through MEK and JNK pathways, and Treg differentiation can be suppressed at least partly through suppressing the activation of ERK and JNK (34, 35). Foxp3 expression can be reduced *via* the activation of TAK1/MKK4/JNK/Smad axis to further induce autophagy (36). In addition, during sepsis, adenosine promotes the expression of Foxp3 in Treg cells through JNK/AP-1 pathway, while JNK-phospho-c-JUN (ser63/73) pathway plays an important role in Foxp3 nuclear translocation in psoriasis (37, 38). In present study, we found that the function of IGFBP5 in regulating Th17/Treg balance could be inhibited by the inhibitors of ERK or JNK pathway. In conclusion, all these data showed that IGFBP5 could regulate Th17/Treg balance *via* ERK or JNK pathways.

DSS-induced UC model was used for *in vivo* experiment. UC is a colonic disease characterized by idiopathic as well as long-lasting inflammation (39). The inflammation of UC is mainly restricted to mucosa surface and submucosa (40). The precise cause of UC is currently unknown, but the genetic susceptibility and dysfunction of mucosal immune response to the gut microbiota are the main factors for the disease occurrence (39, 41). Recent studies have shown that the abnormal innate and adaptive immune response, and the imbalance of intestinal flora, may cause intestinal inflammation in UC patients (42, 43). Among numerous immune cells, Th17/Treg balance is reported to be significant to the immune homeostasis of cellular niche in colon (44, 45). Basing on theses character, DSS-induced UC model was an optional model for research on immune-mediated inflammatory diseases (46, 47).

The balance of Th17/Treg was also critical important for other chronic inflammatory diseases. Th17 cells play important roles in the progression of periodontitis, which makes this group of diseases have something in common in pathogenesis and treatment (48). Our data showed that after the treatment of recombinant IGFBP5, the colon shortening was reversed significantly. In addition, the results of histological examination and HAI indicated that IGFBP5 treatment alleviated colon damage and maintain the stability of intestinal epithelial structure in mice with DSS-induced colitis. Besides, there was an increased number of Th17 cells and a decreased number of Treg cells in colon tissue in mice with DSS-induced colitis, and IGFBP5 reversed the Th17/Treg imbalance, indicating an improvement in DSS-induced colitis. Increased intestinal permeability and immune cell infiltration are considered to improve the amount of mucosal pro-inflammatory cytokines produced by epithelial and immune cells. DSS increases the production of pro-inflammatory mediators TNF-α, IL-1β, IL-6, IFN-γ and chemokine KC and macrophage inflammatory protein-2, which not only play important role in the pathogenesis of DSS-induced colitis, but also serve as crucial intervention targets for colitis (49–52). Our results of ELISA demonstrated that the serum levels of TNF-α, IL-1β and IFN-γ were increased in mice with DSS-induced colitis, but down-regulated after treatment with recombinant IGFBP5. The imbalance of intestinal microbiota is also closely associated with UC. In addition, the result of PCA showed that the composition of the normal group, DSS+PBS group and DSS+IGFBP5 group had some degree of separation, identifying the variation upon IGFBP5 treatment. According to literature reports, the total abundance of mucolytic bacteria increased, while the number of Akkermansia decreased in intestines of patients with IBD, indicating that Akkermansia may have anti-inflammatory effect (53). Similarly, Early et al. showed that the enrichment of Akkermansia in patients with UC decreased, which was negatively correlated with inflammation (54). Vigsnaes et al. suggested that changes in the composition of Gram-negative bacteria, including the reduction of lactobacilli and Akkermansia muciniphila may play an important role in UC (55). Additionally, Dunn et al. revealed that the predominant strains of patients within sustained remission contained a large number of Akkermansia muciniphila after nutritional treatment of IBD (56). However, there is still controversy about the beneficial effects of Akkermansia bacteria, since Ring et al. have shown that Akkermansia muciniphila strain does not accelerate short-term intestinal inflammation in gnotobiotic IL-10-deficient mice (57). Currently, most of the research on Akkermansia are experimental studies focused on the differential expression in the disease, lacking more in-depth research as well as high-quality clinical research. In recent years, the clinical research on Akkermansia bacteria in IBD, metabolic diseases and mental diseases is being carried out effectively, which may provide novel ideas for the therapy of inflammatory-related diseases. To sum up, the therapeutic effect of IGFBP5 on the maintenance of immune homeostasis, the improvement of lymphocyte infiltration and the regulation bacterial flora balance can be used as a reference for the therapy of other immune-mediated inflammatory diseases, including periodontitis and rheumatoid arthritis.

In summary, our study showed that recombinant IGFBP5 could maintain immune homeostasis by down-regulating Th17 cells and up-regulating Treg cells in mice with DSS-induced colitis, which in turn inhibits the inflammatory progression, attenuates disease activity, cells and decreases the production of pro-inflammatory cytokines such as TNF-α, IL-1β and IFN-γ. The results also proved that IGFBP5 regulated gut microbiota in DSS-induced colitis. The effect of IGFBP5 on immuno-inflammatory responses may be achieved partly by affecting Th17/Treg balance through ERK or JNK pathway. More animal models are warranted to provide a theoretical basis for the application of IGFBP5 in the treatment of inflammatory diseases.

## Data availability statement

The datasets presented in this study can be found in online repositories. The names of the repository/repositories and accession number(s) can be found below: Sequence Read Archive (SRA) database under the accession number PRJNA893336.

## Ethics statement

The animal study was reviewed and approved by the Animal Ethical and Welfare Committee of Capital Medical University School of Stomatology.

## Author contributions

MZ drafted this manuscript. MZ and HH performed the experiments, analyses, and interpretation of the data. LH, YC and ZF helped the aforementioned authors to develop the experiments. YC and ZF provided final approval of the version to be published. All authors contributed to the article and approved the submitted version.

## Funding

This work was supported by CAMS Innovation Fund for Medical Sciences (2019-I2 M-5- 031 to ZF), the Program for ‘Hundred-Thousand- Ten Thousand’ Talents in Beijing (2018A16 to ZF), Beijing Key Laboratory of Tooth Regeneration and Function Reconstruction (KFKT2019012 to LL) and grants from Innovation Research Team Project of Beijing Stomatological Hospital, Capital Medical University (NO.CXTD202204 to ZF).

## Conflict of interest

The authors declare that the research was conducted in the absence of any commercial or financial relationships that could be construed as a potential conflict of interest.

## Publisher’s note

All claims expressed in this article are solely those of the authors and do not necessarily represent those of their affiliated organizations, or those of the publisher, the editors and the reviewers. Any product that may be evaluated in this article, or claim that may be made by its manufacturer, is not guaranteed or endorsed by the publisher.
