# Supplementary material for: Insulin-binding protein-5 down-regulates the balance of Th17/Treg
Source: Front Immunol. 2022 Nov 1;13:1019248. doi: 10.3389/fimmu.2022.1019248 (PMC9664073; doi:10.3389/fimmu.2022.1019248)
Supplement: Supplementary file 1 [file DataSheet_1.docx]

***Supplementary Material***

**Supplementary Figures**


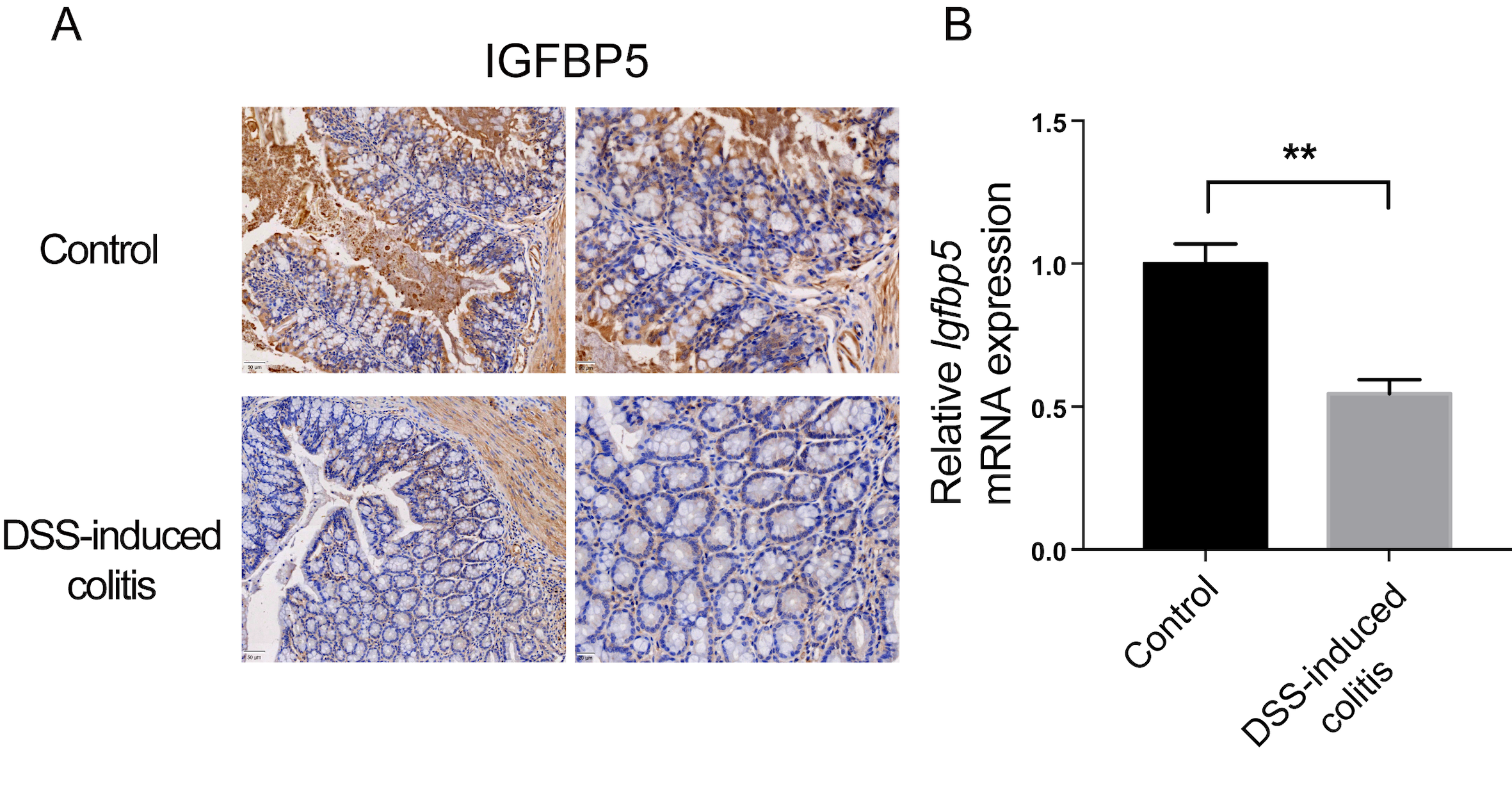


**Supplementary Figure 1. IGFBP5 expression reduced in mice with DSS-induced colitis. (A)** IGFBP5 expression in colon tissues of control and mice with DSS-induced colitis by immunohistochemical staining (upper: normal colon tissue; lower: colon tissue of mice with DSS-induced colitis. Left: 20×; right: 40×). **(B)** IGFBP5 mRNA expression in colon tissues of control and mice with DSS-induced colitis by RT-qPCR. Unpaired t-test was used to determine statistical significance. The data are presented as the mean ± SEM (n=5). The experiment was repeated three times independently. ***p*<0.01
